# Predictors of decline in walking ability in community-dwelling Alzheimer’s disease patients: Results from the 4-years prospective REAL.FR study

**DOI:** 10.1186/alzrt216

**Published:** 2013-10-29

**Authors:** Yves Rolland, Christelle Cantet, Philipe de Souto Barreto, Matteo Cesari, Gabor Abellan van Kan, Bruno Vellas

**Affiliations:** 1 INSERM, U1027, F-31073 Toulouse, France; 2 University of Toulouse III, F-31073 Toulouse, France; 3 Gerontopole, Toulouse University Hospital, Toulouse, France; 4 Service de Médecine Interne et de Gérontologie Clinique, Pavillon Junot, 170 avenue de Casselardit. Hôpital La Grave-Casselardit, Toulouse, France

## Abstract

**Introduction:**

The aim of this study was to explore the predictors of decline in walking ability in patients with Alzheimer’s disease (AD).

**Methods:**

The prospective REseau surla maladie ALzheimer FRançais (REAL.FR) study enrolled six hundred eighty four community-dwelling AD subjects (71.20% women; mean age 77.84 Standard Deviation, SD, 6.82 years, Mini-Mental State Examination 20.02, SD 4.23). Decline in walking ability was defined as the first loss of 0.5 points or more in the walking ability item of the Activities of Daily Living scale (ADL), where higher score means greater independence, during the four-years of follow-up. Demographic characteristics, co-morbidities, and level of education were reported at baseline. Disability, caregiver burden, cognitive and nutritional status, body mass index, balance, behavioral and psychological symptoms of dementia, medication, hospitalization, institutionalization and death were reported every six months during the four years. Cox survival analyses were performed to assess the independent factors associated with decline in walking ability.

**Results:**

The mean incident decline in walking ability was 12.76% per year (95% Confidence Interval (CI) 10.86 to 14.66). After adjustment for confounders, the risk of decline in walking ability was independently associated with older age (Relative Risk, RR = 1.05 (95% CI 1.02 to 1.08)), time from diagnosis of dementia (RR = 1.16 (1.01 to 1.33)), painful osteoarthritis (RR = 1.84 (1.19 to 2.85)), hospitalization for fracture of the lower limb (RR = 6.35 (3.02 to 13.37)), higher baseline ADL score (RR = 0.49 (0.43 to 0.56)), and the use of acetylcholinesterase inhibitors (RR = 0.52 (0.28 to 0.96)).

**Conclusions:**

The risk of decline in walking ability is predicted by older age, increased dementia severity, poor functional score, and orthopedic factors and seems to be prevented by the use of acetylcholinesterase inhibitors medication.

## Introduction

Longitudinal studies of subjects with Alzheimer’s disease (AD) have mainly explored the gradual decline of cognitive performance and the changes in behavioral domains. The decline of ability to perform activities of daily living, such as walking, has received less attention. However, AD is also characterized by an early deterioration in mobility [1] of significant practical importance [2-4].

Mobility limitation in AD patients results in approximately two or three times higher risk of falling than a similar mobility limitation in cognitively intact older adults [5]. Moreover, the ability to walk is also a key determinant of an AD patient’s quality of life [6], institutionalization [7], risk of death [8] and burden for the caregiver and the community [9].

Numerous factors may explain the decline in walking ability during the course of AD. Poor executive function has been associated with poor physical performance. In severe AD patients, extrapyramidal symptoms can be observed [5,10,11], potentially leading to fall and fracture events. Psychotropic treatment, weight loss, depressive symptoms [12], behavior disturbance and poor balance due to prolonged bed-rest, may also be associated with gait disorders and loss of walking ability. To our knowledge, no study has previously reported the longitudinal change in mobility using the walking item of the Activities of Daily Living (ADL) scale in people with AD and investigated their predictors in community-dwelling AD patients.

The identification of risk factors associated with future mobility impairment represents a first step for the optimization of preventive intervention in this vulnerable population. Moreover, given the widespread use and clinical relevance of the ADL scale, the present analyses may provide results that may be easily implemented in routine clinical practice. In fact, by showing that specific risk factors may predict future decline of walking ability in AD patients, we may hypothesize about the design of effective interventions aimed at reversing this deterioration. Thus, the aim of the present prospective study is to investigate predictors of decline in walking ability in a large cohort of AD patients, followed-up for four years.

## Methods

### Study participants

We conducted secondary analyses of a large prospective epidemiological study, the REAL.FR study (REseau sur la maladie ALzheimer FRançais). The sampling and data collection procedures have been previously described in detail [13].

Briefly, from 2000 to 2002, 686 out-patients with AD were enrolled. This study was carried out in 16 university hospital departments of neurology, geriatrics or psychiatry in 10 French cities to investigate the natural history of AD. For inclusion, subjects had to meet the National Institute of Neurological and Communicative Diseases and Stroke/Alzheimer Disease and Related Disorders Association (NINCDS-ADRDA) criteria for probable or possible AD [14]. Additionally subjects were required to be community-dwelling and to have a caregiver who was willing to participate in the study. Patients with a diagnosis of an illness with a life expectancy less than 24 months were excluded. Subjects were assessed at an outpatient consultation every six months for four years. For the current study, two participants were excluded from the analyses because of baseline disability with walking as recorded in the relevant ADL item. The REAL.FR study was supported by French Ministry of Health Grants (PHRC 98-47 N and PHRC 18-05) and received ethical approval from the Advisory Committee for the Protection of Persons participating in Biomedical Research at the Toulouse University Hospital. It was conducted as an observation investigation and no signed informed consent was required.

### Decline of walking ability

The ADL scale [15] was used to evaluate subjects’ functional status. The six basic ADL items include: eating, walking, continence, using the toilet, bathing and dressing. The items are scored 0 (unable to do), 1 (do without any human assistance) or 0.5 (need for assistance). Decline of walking ability was defined as the first loss of 0.5 points or more on the ‘walking ability’ item during follow-up. Scoring was performed after examination of the patient and direct inquiry with the caregiver every six months in the day hospital over a four-year period.

### Covariates

Age, gender and length of time since diagnosis were reported. Caregiver and participants were asked whether the subject had (yes/no) any of the following cardiovascular risk factors (diabetes mellitus, past or current smoking, hypertension, dyslipidemia), cardiovascular disease (atrial fibrillation, angina, heart failure, myocardial infarction, peripheral vascular disease), musculoskeletal and painful osteoarthritis (back and/or legs) or other co-morbidities (respiratory, sensorial, gastro-intestinal, kidney, urinary, neurological or endocrine disease). The presence of depression was obtained by direct inquiry with the caregiver. Level of education (illiterate, elementary, primary, high school or post-graduate degree) was recorded. At baseline, a cerebral tomography (CT-scan) was performed and independently interpreted by both a radiologist and a neurologist as follows: normal, atrophy alone, lacuna or stroke or hypodensity of the white matter or other vascular lesion. CT-scans were reassessed by both assessors in the case of conflicting interpretation.

Mortality was reported at the clinic visit every six months over the four years and was ascertained by telephone call to caregivers and/or primary-care physicians, if necessary.

Some covariates considered in the present analyses were computed as time-dependent variables: disability, caregiver burden, cognitive and nutritional status, body mass index, balance, behavioral and psychiatric symptoms of dementia (BPSD), medication, hospitalization, and institutionalization were reported at the clinic visit every six months.

Functional status was defined as the sum of the ADL score excluding the walking item. Scores ranged from 0 to 5, with a lower score meaning worse function. The ADL score was investigated as an independent variable since walking ability modification may be affected by the overall baseline functional status. Caregiver burden was assessed using the Zarit scale [16]. Dementia severity was assessed using the mini-mental status examination (MMSE <20, 17 to 22 or 22 to 27) [17], behavioral and psychiatric symptoms of dementia (BPSD) were assessed using the Neuro-Psychiatric Inventory (NPI) [18], and nutritional status was evaluated using the Mini-Nutritional Assessment (MNA) [19]. Body mass index (BMI = weight in kilograms/squared height in kilogram meters) was also calculated. Balance was assessed using the ‘one-leg balance’ test described by Vellas *et al*. [20]. The test was performed by asking the subject to stand unassisted on one leg as long as possible (eyes open, barefoot or not, using whichever leg was spontaneously chosen by the subject). The ‘one-leg balance’ test was reported as abnormal when the subject was unable to stand on one leg for five seconds or more [20]. The test was performed twice with the best result used for the analysis. Caregivers were asked about the patient’s current medication. Additionally, at assessment interview, subjects were asked to bring with them all their regular medication. Prescriptions of acetylcholinesterase (AChE) inhibitors (galantamine, donepezil or rivastigmine), psychotropic treatments (anxiolytics, neuroleptics, serotonin reuptake inhibitors and other antidepressants), anti-Parkinsonian treatment (dopamine agonist or dopaminergic agent) and vitamin D were collected and used as additional covariates. NMDA-receptor antagonist (memantine) was not available in France at that time, and, thus, not considered for the present analyses. Subjects and caregivers were asked about nursing-home admission and hospitalization. The reason for hospitalization was recorded. Subjects who did not attend follow-up appointments were contacted by telephone and post if necessary.

### Statistical analysis

Baseline characteristics were described using mean values ± standard deviation (SD) and proportions for quantitative and qualitative variables, respectively. Survival analyses (Cox models) were performed to identify independent predictive factors of decline in walking ability, using relative risk (RRs) and 95% confidence interval (CIs). Patients were followed up until the first occurrence of the event (decline in walking ability) or until the censor date (drop-out, death, final endpoint). For time-dependent variables (disability, caregiver burden, cognitive and nutritional status, body mass index, balance, behavioral and psychiatric symptoms of dementia (BPSD), medication, hospitalization and institutionalization), survival analyses were based on the measure collected during the visit preceding the event, except for hospitalization and institutionalization which were considered regardless of the period when they occurred [21]. The AChE inhibitor treatment (or the absence of treatment) was taken into account during at least the six-month period preceding the decline in walking ability. For time-independent variables, analyses were based on the measure collected at baseline. Cox proportional hazards models with discrete times (because decline in walking ability occurs between the follow-up visits) using a backwards selection procedure were performed with *P* <0.20 as an entry criterion and *P* >0.05 as a removal criterion. Two models (with or without the ADL introduced in the initial model) were performed due to the fact that the ADL score is strongly correlated with walking ability and decline in walking ability may be both the cause and/or the consequence of the ability to perform basic motor tasks. Tests based on interaction with time were used to ascertain the proportional hazards assumption for time-constant variables (age, gender, level of education, length of time from diagnosis of dementia, cardiovascular risk factors, cardiovascular disease or other co-morbidities, CT). Statistical interactions were verified in the final model. *P*-values were based on two-sided tests. All statistical analyses were performed using SAS software (version 9.3, SAS Institute Inc. Cary, NC, USA).

## Results

**Figure 1:**
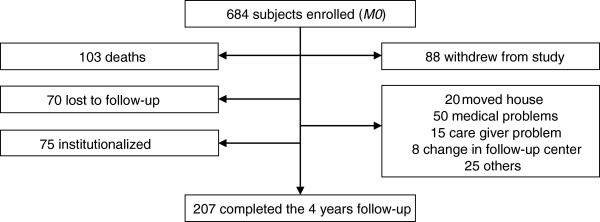
**Flow chart of the REAL.FR population study during the four years of follow-up.** REAL.FR, REseau sur la maladie ALzheimer FRançais.

The flow chart of the four-year REAL.FR prospective study is shown in Figure 1. Among the 684 AD subjects recruited at baseline, 207 attended the final follow up visit at four years. During this period of observation, 103 participants died, 88 declined continuation in the study, 75 were institutionalized and 70 were lost to follow-up. Compared to the patients followed over the four years, the patients who did not completed the four-year follow up (Figure 1) were, at baseline, significantly older (78.51 years; SD 6.74 versus 76.53; SD 6.81), were less frequently women (68.72% versus 76.09%), were more disabled (ADL = 5.33; SD 0.96 versus 5.67; SD 0.60), more severely demented (MMS = 19.56; SD 4.34 versus 20.94; SD3.84), had more behavior disturbance (NPI = 16.70; SD 16.35 versus 12.69; SD 12.57), had more cardiovascular disease (29.52% versus 17.84%) and had more frequently an abnormal one-leg balance test (19.17% versus 7.31%) (all *P* values <0.05). Baseline characteristics of the study sample are summarized in Table 1. The incidence decline in walking ability was 12.76% (95%CI, 10.86% to 14.66%) per year during the four years of follow-up.

**Table 1:** Baseline characteristics of the subjects (n = 684)

**Variables**	**Values**
Age (mean, SD, years)	77.84 (6.82)
Gender, female (%)	71.20
Level of education (% > primary school)	78.79
Age at diagnosis (mean, SD, years)	76.77 (7.00)
Cardiovascular risk factors^a^ (%)	59.65
Cardiovascular disease (%)	25.59
Painful osteoarthritis (%)	8.50
Depression (%)	37.17
Comorbidity (%)	
0	28.09
1	37.96
≥ 2	33.95
MNA (% ≤ 23.5)	33.03
Zarit score (mean, SD)	22.63 (15.87)
MMSE (mean, SD)	20.02 (4.23)
MMSE (%)	
> 20	50.44
16 to 20	32.55
< 15	17.01
One leg balance test (% abnormal)	15.06
ADL score (mean, SD)	5.45 (0.87)
Weight (kg, mean, SD)	62.63 (12.76)
BMI (%)	
< 20	13.33
20 to 25	43.26
25 to 30	33.63
≥ 30	9.78
NPI (mean, SD)	15.35 (15.30)
NPI (%)	
Delusions	16.23
Hallucination	6.73
Agitation/aggression	36.99
Depression	38.74
Anxiety	42.25
Elation/euphoria	7.02
Apathy	53.51
Disinhibition	10.96
Irritability/lability	36.26
Aberrant motor behavior	21.78
Sleep disorder	14.62
Appetite disorder	22.11
Treatment (%)	
AChE inhibitors	89.33
Psychotropic treatments	50.15
Anti-Parkinsonian treatment	1.32
Computed tomography scan (%)	
Normal	22.58
Atrophy alone	26.69
Lacuna or stroke	9.68
Hypodensity of the white matter	12.17
Other	8.65
CT scan not performed	20.23

^a^Cardiovascular risk factors, diabetes mellitus, past or current smoking, hypertension, dyslipidemia, cardiovascular disease, atrial fibrillation, angina, heart failure, myocardial infarction, peripheral vascular disease; comorbidity, respiratory, sensorial, gastro-intestinal, kidney, urinary, neurological disease, endocrine, alcohol and other; anti-parkinsonian treatment, dopamine agonist or dopaminergic agent; psychotropic treatments, anxiolytics, neuroleptics, serotonin reuptake inhibitor and other antidepressants; AChE inhibitors, acetylcholinesterase (AChE) inhibitors (galantamine, donepezil or rivastigmine); ADL, Activities of Daily Living scale; BMI, body mass index; MNA, Mini-Nutritional Assessment, ≤23.5 versus >23.5; MMSE, Mini-Mental State Examination; NPI, Neuro-Psychiatric Inventory.

In the first model (Table 2), (after adjustment for confounders without the variable ADL), the following independent factors remained significantly associated with decline in walking ability: older age (RR = 1.05 (1.02 to 1.08)), length of time from diagnosis of dementia (RR = 1.20 (1.05 to 1.37)), painful osteoarthritis (RR = 1.81 (1.17 to 2.78)), MNA score (RR = 1.45 (1.01 to 2.08)), institutionalization (RR = 1.83 (1.03 to 3.25)), hospitalization for lower limb fracture (RR = 5.80 (2.85 to 11.81)), MMSE score between 16 to 20 versus >20 (RR = 1.07 (0.66 to 1.74)) and MMSE score <15 versus >20 (RR = 2.41 (1.54 to 3.75)), abnormal one leg balance test (RR = 2.25 (1.44 to 3.51)) and use of AChE inhibitors (RR = 0.48 (0.26 to 0.89)) (Table 2). In the second model (after adjustment for all confounders including the variable ADL), the following independent factors predicted the decline of the ability to walk: older age (RR = 1.05 (1.02 to 1.08)), length of time from diagnosis of dementia (RR = 1.16 (1.01 to 1.33)), painful osteoarthritis (RR = 1.84 (1.19 to 2.85)), hospitalization for lower limb fracture (RR = 6.35 (3.02 to 13.37)), higher ADL score (walking item excluded) (RR = 0.49 (0.43 to 0.56)) and use of AChE inhibitors remained in this final model (RR = 0.52 (0.28 to 0.96)).

**Table 2:** Univariate and multivariate logistic regression analyses of factors associated with the decline of walking ability

	**Relative risk (95% confidence interval)**		
	**Univariate**	**Stepwise backward regression analysis**	**Stepwise backward regression analysis**
		**Model 1**	**Model 2**
Age	1.06 (1.04 to 1.09)	1.05 (1.02 to 1.08)	1.05 (1.02 to 1.08)
Gender (reference group is male)	0.78 (0.54 to 1.12)	-	-
Length of time since the diagnosis of dementia	1.14 (1.01 to 1.28)	1.20 (1.05 to 1.37)	1.16 (1.01 to 1.33)
Cardiovascular risk factors	1.22 (0.88 to 1.70)	-	-
Cardiovascular disease	1.59 (1.11 to 2.27)	-	-
Painful osteoarthritis	1.62 (1.11 to 2.37)	1.81 (1.17 to 2.78)	1.84 (1.19 to 2.85)
Comorbidity			
0	1	-	-
1	1.07 (0.71 to 1.61)		
≥ 2	1.52 (1.01 to 2.29)		
MNA (≤23.5 versus >23.5)	2.01 (1.47 to 2.74)	1.45 (1.01 to 2.08)	-
Institutionalization (yes versus no)	2.89 (1.77 to 4.73)	1.83 (1.03 to 3.25)	-
Hospitalization for fracture of the lower extremity (yes versus no)	4.27 (2.34 to 7.80)	5.80 (2.85 to 11.81)	6.35 (3.02 to 13.37)
Hospitalization for stroke (yes versus no)	2.14 (0.62 to 7.37)	-	-
Hospitalization for reason other than fracture or stroke (yes versus no)	1.62 (1.11 to 2.35)	-	-
MMSE			
> 20	1	1	-
16 to 20)	1.36 (0.89 to 2.06)	1.07 (0.66 to 1.74)	
< 15	3.02 (2.06 to 4.44)	2.41 (1.54 to 3.75)	
One leg balance test (abnormal versus normal)	3.10 (2.13 to 4.51)	2.25 (1.44 to 3.51)	-
ADL score (walking item excluded)	0.49 (0.43 to 0.55)	Not introduced in the model 1	0.49 (0.43 to 0.56)
BMI		-	-
< 20	1.22 (0.73 to 2.04)		
20 to 25	1		
25 to 30	1.03 (0.72 to 1.47)		
> 30	1.76 (1.09 to 2.82)		
Hallucination (yes versus no)	1.63 (0.94 to 2.86)	-	-
Apathy (yes versus no)	1.70 (1.23 to 2.37)	-	-
Aberrant motor behavior (yes versus no)	1.74 (1.27 to 2.39)	-	-
AChE inhibitors (yes versus no)	0.41 (0.25 to 0.66)	0.48 (0.26 to 0.89)	0.52 (0.28 to 0.96)
Psychotropic treatments (yes versus no)	1.36 (0.99 to 1.88)	-	-
Anti to Parkinsonian treatment (yes versus no)	2.32 (0.79 to 6.80)	-	-

Anti-parkinsonian treatment: dopamine agonist or dopaminergic agent; psychotropic treatments: anxiolytics, neuroleptics, serotonin reuptake inhibitor and other antidepressants. AChE inhibitors, acetylcholinesterase inhibitors (rivastigmine or donepezil or galantamine); ADL, activities of daily living score with walking item excluded (score over 5); BMI, body mass index; MNA, Mini-Nutritional Assessment <23.5 versus ≥24; MMS score, Mini-Mental state score.

## Discussion

This prospective study reports a high rate of decline in walking ability in a community-dwelling AD population. In this population, we reported a 12.76% annual incidence of walking ability decline. After four years, about one third (30.20%) of the remaining sample needed assistance or had lost the ability to walk. We also show that high risk of decline in walking ability is associated with age, time since diagnosis of dementia, poor baseline functional score and orthopedic factors but treatment with AChE I appears protective.

The ability to walk has been reported to be a major factor affecting quality of life in patients with dementia [6]. Once a patient with AD has lost their ability to walk, he/she is generally unable to regain it [22]. Delaying loss of the ability to walk during the disease course for as long as possible should be a goal in care planning. Previous studies have reported the significant changes that occur in gait characteristics and gait speed [1]. Physical performance tests [23] and specific scales, for example the Unified Parkinson's Disease Rating Scale (UPDRS), have been used to measure the changes in motor signs in people with AD in longitudinal studies [24]. We believe that the reported rate of decline in walking ability during a four-year follow-up, using the ADL score, provides clinically relevant data for potential interventional studies aimed at slowing the decline in walking ability.

Age, time since diagnosis of dementia and poor baseline functional score were expected results from this study. Osteoarthritis [25] and hip fracture [26] are also well-known and strong risk factors for disability, especially for the mobility domain and in the context of dementia [27].

AChE inhibitors were found to be associated with a lower risk of decline in walking ability and this factor remained statistically significant in the different models. A statistically significant improvement in global function of patients treated by AChE inhibitors was previously reported in some studies [28-30] but not in others [31]. Reviews and meta-analyses on the beneficial effect of the AChE inhibitors on global functioning support only a modest benefit in slowing decline in global function [32]. These previous studies have addressed all ADL domains that include heterogeneous items (urinary incontinence, transfer, dressing, eating and walking). Then, from an analytic perspective an improvement in just one area might be clinically significant but statistically masked by no effect in other functional domains. The previous randomized clinical trials on global functional score do not allow specific conclusions on the ability to walk.

In our study, the suggested protective effect of AChE inhibitors on decline in walking ability raises several hypotheses. Maintaining the ability to walk relies on many factors, including cognitive function, balance and strength but also physical training, behavior disturbance and environment. Some of these factors can be, at least in part, directly or indirectly improved by AChE inhibitors. AChE inhibitors have demonstrated a positive effect on cognition [33,34] and studies suggest that galantamine may more specifically improve attention and executive function [35]. Since AChE inhibitors improve gait velocity and reduce gait variability [36], it is plausible to think that participants on AChE inhibitors may develop a better gait strategy improving their ability to adapt their gait pattern to unexpected situations, hence reducing their risk of falls [36]. Moreover, AChE inhibitors may impact walking ability indirectly through a reduction in behavioral disturbance. Indeed, AChE inhibitors have positive effects on a variety of behavioral and psychiatric symptoms in AD, particularly for apathy, depression and anxiety [37]; these symptoms are often associated with low physical activity and physical deconditioning, which may impact on the ability to walk in the longer-term. The effect of AChE inhibitors on peripheral neuromuscular mechanisms has been poorly explored compared to the central effect and requires further study and validation. However, the cholinergic neuromuscular junction of skeletal muscle is potentially a rich source of amyloid-beta (Aβ) peptides and AD might have systemic manifestations [38]. Moreover, AChE inhibitors can generate fasciculation in rats [39] and experimental electrophysiological data support an improvement in the neuro-muscular properties of AD patients treated with AChE inhibitors [40]. Finally, AChE inhibitors may attenuate the negative effect of the systemic inflammatory responses through direct or indirect mechanisms. Increased peripheral concentrations of inflammatory markers have been reported in AD patients [41], and acetylcholine can inhibit the production of pro-inflammatory cytokines [42]. Basic research supports the theory that donepezil has neuroprotective and cardioprotective effects via anti-inflammatory mechanisms but other organs, such as the muscle, may be affected [43].

This study has several limitations. First, discontinuation of AChE inhibitors is a frequent event and may be related to the acceleration of the functional decline. Cognitive decline, anxiety, weight loss and hospitalization have been reported in this cohort to predict discontinuation of the AChE inhibitors [44]. To reduce this limitation (AChE inhibitors discontinuation may be the consequence of the functional decline or the decline of the walking ability), we took into account discontinuation of the AChE inhibitors at least six months prior to the decline in walking ability. Despite this, AChE inhibitors remained significantly associated with the decline in walking ability in the final model which included the ADL score. However, we cannot ignore the fact that treatment with AChE inhibitors was only a marker of better walking ability. Second, many factors, such as pain, low level of physical activity and deconditioned balance because of prolonged bed-rest, were not assessed in our study and may account for the decline in walking ability. However, hospitalization and painful osteoarthritis were taken into account in our analyses. Third, selection bias could also have occurred because the recruitment of the participants was performed via specialized memory centers in France. The attrition rate was high although similar to other cohorts of patients with dementia. Characteristics of the patients lost to follow up compromise the generalization of our results. However, patients who refused to participate, were institutionalized or lost to follow up may have experienced a higher rate of functional decline, probably leading to the under-estimation of incident walking impairment. Finally, the choice of the ADL scale to assess the ability to walk is open to criticism. The ADL is useful in the management of AD but the walking item may not be sensitive to small but significant changes. Additional data on physical performance such as the gait speed test would have reinforced our results.

## Conclusions

In conclusion, our results suggest that decline in walking ability is associated with age, time since diagnosis of dementia, overall functional status and orthopedic factors. It is reduced by current prescription of AChE inhibitor medication. Future studies should investigate whether the benefit of AChE inhibitors on the ability to walk may be reproduced and confirmed in *ad hoc* designed interventional trials.

## Abbreviations

AChE: Acetylcholinesterase; AD: Alzheimer’s disease; ADL: Activities of Daily Living Scale; BMI: Body mass index; BPSD: Behavioral and psychiatric symptoms; CI: Confidence interval; CT-scan: Cerebral tomography; MMSE: Mini-mental Status Examination; MNA: Mini-nutritional Assessment; NINCDS-ADRDA: National Institute of Neurological and Communicative Diseases and Stroke/Alzheimer Disease and Related Disorders Association; NMDA-receptor: N-methyl-D-aspartate receptor; NPI: Neuro-Psychiatric Inventory; PHRC: Projet hospitalier de recherche Clinique; REAL.FR: REseau sur la maladie ALzheimer FRançais; RR: Relative risk; SD: Standard deviation.

## Competing interests

The authors declare that they have no competing interests.

## Authors’ contributions

YR, PdSB, MC, GAvK and BV contributed to the conception of the study and the writing of the manuscript. CC contributed to analysis and writing of the manuscript. All authors read and approved the final manuscript.
